# The Role of Phonological, Auditory Sensory and Cognitive Skills on Word Reading Acquisition: A Cross-Linguistic Study

**DOI:** 10.3389/fpsyg.2020.582572

**Published:** 2020-11-12

**Authors:** Cristina Ferraz Borges Murphy, Eliane Schochat, Doris-Eva Bamiou

**Affiliations:** ^1^The Ear Institute, University College London, London, United Kingdom; ^2^Department of Physical Therapy, Speech-Language Pathology and Occupational Therapy, School of Medicine, University of São Paulo, São Paulo, Brazil; ^3^Department of Neuro-Otology, University College London Hospitals, London, United Kingdom; ^4^Biomedical Research Centre, National Institute for Health Research, London, United Kingdom

**Keywords:** reading, phonological awareness, auditory processing, attention, memory, cross-linguistic

## Abstract

Despite considerable evidence regarding the influence of orthography on reading processing, the impact of orthographic depth on reading predictors remains unclear. In addition, it also remains unknown the role of the orthography in the influence of auditory temporal processing and attention skills on word reading skills. The current study investigates the word reading predictors in a group of British and Brazilian children with typical development considering phonological, auditory sensory, short-term memory, and sustained attention skills. Rhyme and Alliteration skills predicted word reading in both groups; however, the correlation in the British group was more robust. Short-term memory was also correlated with reading in both groups; however, it was a significant word reading predictor only in the British group. The auditory sensory was not directly correlated with word reading in either group; however, it was involved with Rhyme and Alliteration performance only in the British group. Those results were discussed considering the complexity of the phonological structure and opaque orthography in English when compared to Portuguese, which indicates that the less transparent the orthography, the higher the importance of factors such as phonological awareness, short-term memory, and to some extent, auditory sensory processing skills on word reading acquisition. Those results emphasize the need to consider orthography and phonological features of a particular language when developing a reading assessment and treatments.

## Introduction

Research has shown that word reading is an important predictor for reading fluency in the first years of formal education (Clemens et al., 2011; Morris and Perney, 2018). As observed by Ehri (2005, 2014), it involves automatically and unintentionally either the process of decoding or reading from memory by sight, depending on the child’s stage of reading and word familiarity. Beginner readers, for instance, are likely to rely on grapheme-phoneme conversion; in contrast, skilled readers could identify most of the printed words through whole-word recognition or even use a combination of both strategies depending on word familiarity.

Studies have also demonstrated that using those word reading strategies can also be affected by orthographic transparency and phonological structure of words in alphabetical languages (Katz and Frost, 1992; Ziegler and Goswami, 2005; Goswami, 2008; Ziegler et al., 2010). For instance, in languages such as Italian and Spanish, not only the spelling-sound correspondence is highly consistent (transparent orthography), but also the predominant phonological structure of syllables is simple (consonant-vowel), enabling the successful use of grapheme-phoneme correspondence as the main strategy when learning to read (Goswami, 2008). The use of this strategy decreases as reading is established, and words can progressively be read from memory by sight (Share, 2008). On the other hand, in opaque languages with phonologically complex syllables, such as English, the alphabetic system is variable and open to decoding errors; therefore, children are required to use decoding strategies involving different grains sizes (sound units), such as the whole-word strategy and rhyme analogy to read the significant number of irregular words and acquire reading proficiency (Ziegler and Goswami, 2005). The use of those strategies seems to persist for longer than in transparent orthographies (Deacon and Kirby, 2004).

Despite considerable evidence regarding the influence of orthography on reading processing, the impact of orthographic depth on reading predictors remains unclear. Ziegler et al. (2010) investigated the influence of phonological awareness, memory, vocabulary, rapid naming, and non-verbal intelligence in several languages varying in orthography transparency. The results showed that phonological awareness was the main predictor of reading accuracy in all languages, except in Finish, the most transparent orthography studied. The influence varied according to the level of transparency, with strong correlations for the most opaque orthography (French). Phonological short-term memory, on the other hand, predicted reading accuracy in both most transparent orthographies (Finish and Hungarian). Vaessen and Blomert (2010) investigated the influence of cognitive skills, including working memory, on reading development in Hungarian, Dutch, and Portuguese. Their results indicated a similar pattern of results in all three orthographies, with working memory being a modest or non-significant predictor for reading in all. Caravolas et al. (2012) also investigated the impact of phoneme awareness and verbal memory span in different orthographies and showed that both skills had the same relative importance regardless of the language.

Those studies investigating the influence of orthography on reading predictors have mostly focused on phonological processing, memory, and rapid automated naming. However, studies involving children with reading difficulties have shown that some other factors involving both top-down and auditory sensory aspects might also be relevant to the reading process (Tallal et al., 1993; Willcutt et al., 2000, 2005; Hämäläinen et al., 2013; Goswami et al., 2014; Gokula et al., 2019). Several studies have shown, for instance, the poor performance of children with reading difficulties in tests involving auditory temporal processing skills (Tallal et al., 1993; Van Ingelghem et al., 2001; Murphy and Schochat, 2009a, b; Goswami et al., 2014; Casini et al., 2018; Fostick and Revah, 2018). According to the “rapid temporal processing hypothesis” (Tallal, 1980), for instance, a subgroup of children with language disorders, including dyslexia, would struggle to process and discriminate rapidly transient acoustic elements, such as the rapid formant transitions between stop consonants and vowels. Because this deficit would affect the establishment of an acoustic representation of phonemes, it would also impact the development of phonological and, consequently, literacy skills, given the significant importance of phonological processing for word reading acquisition. According to a second hypothesis, called “temporal sampling framework,” the auditory temporal processing deficit would involve the ability to discriminate and categorize amplitude-modulated sounds in terms of rising time, leading to phonological deficits related to prosodic stress, onset, and rime manipulation (Goswami et al., 2002). Although those links between reading and auditory temporal processing have been extensively studied, the influence of orthographic depth and the use of different reading strategies on this relationship has not been addressed.

Emerging theories have also associated reading and overall attention skills (Casco et al., 1998; Dally, 2006; Shaywitz and Shaywitz, 2008; Gokula et al., 2019), especially through studies addressing the high rate of comorbidity of dyslexia and attention-deficit/hyperactivity disorder (Willcutt et al., 2000; Aaron et al., 2002). Casco et al. (1998), for instance, reported a correlation between attention task performance and reading rate. Dally (2006) demonstrated that inattentive behavior in children in the first year of formal schooling affects the development of phonological analysis abilities, such as the manipulation of sublexical components of words. Sims and Lonigan (2013), investigating the relationship between reading and sustained attention specifically, also linked early reading skills in young children and attention performance, corroborating the idea that attention plays an important role in early reading development skills. Despite that, the role of orthography has not been considered in those studies involving the influence of attention on word reading skills.

The main goal of the current study was to examine the word reading predictors in two languages differing in orthographic transparency and phonological structure. The two specific purposes will be: (1) to investigate whether the importance of phonological processing, including phonological awareness and short-term phonological memory, varies with orthographic transparency, given the controversy regarding the relative influence of phonological processing, and (2) to investigate the role of orthography in the influence of auditory temporal processing and attention skills on word reading skills, given the lack of studies addressing the impact of those factors on different orthographies.

Word reading, phonological awareness, short-term memory, sustained attention, and auditory temporal processing skills were assessed in a group of British and Brazilian children with typical development. Phonological and orthographical differences between the Brazilian Portuguese and the English language are well-established (Azevedo, 1981). The English language has not only more consonants and vowels than Portuguese but also a more significant number of consonants occurrences in spoken language. The words in English have fewer numbers of syllables and more complex syllable structures. Also, both languages have different levels of orthographic consistency, with English being the least consistent in terms of its spelling-to-sound relationships. Given the complex phonological structure and opaque orthography in the English language and, consequently, the frequent use of phonological reading strategies, we hypothesize that the phonological awareness factor would be a stronger predictor of reading in English than Brazilian Portuguese. Consequently, we also hypothetize that the auditory temporal processing and attention skills might also be stronger reading predictors in English, given the importance of phoneme discrimination and attention skills when manipulating phonemes. From a clinical perspective, we expect the results to contribute to a better understanding of universal and specific reading predictors in different languages for a more appropriate assessment and treatment of reading disorders.

## Materials and Methods

This study was conducted at the University College London (UCL) Ear Institute and was approved by the UCL Research Ethics Committee under protocol number 6688/001. It also had the collaboration of the University of São Paulo, where the Brazilian group was collected. A written consent form with detailed information about the aim and the protocols of the study was also approved by this ethics committee. All parents provided written informed consent on behalf of their children prior to participation in the study.

### Participants

A total of 100 typically developing children, aged 5–10 years, took part in this study (Table 1). The Brazilian group was composed of 58 native Brazilian Portuguese speakers and the British group was composed of 42 native English speakers, all monolinguals. The children were enrolled in the first years of primary school, which starts from 5 years old in the United Kingdom and from 6 years old in Brazil. Both groups were taught to read through the Phonics method. The Brazilian children were from the same region of São Paulo city, suggesting a similar socio-economic background and the British children, who were living in the same borough in London. The level of education of the main caregiver was ascertained in both groups.

**TABLE 1:** Characteristics of the participants.

	British group (*n* = 42)	Brazilian group (n = 58)
**Gender (*n*)**		
Girls	47.6%	36.2%
Boys	52.3%	63.7%
**Age (*M* ± *SD*)**	7.68 ± 1.59	7.27 ± 0.77
**Caregiver education (years)**	14.4	15.1
**Auditory Task**		
*Audiological evaluation*	Normal	Normal

The children, from both groups, were required to have no familial or personal history of diagnosed or suspected auditory problems (including any otological disease since birth such as recurring middle-ear disease and listening difficulty such as understanding speech in the presence of background noise), no developmental disorders, speech and language difficulties, psychological or neurological disorder or injuries. Additionally, the participants were required to pass an audiometric screen in a quiet room in their school using headphones (pure-tone thresholds < 30 dB HL at 1, 2, and 4 kHz).

### Tests

#### Cognitive Measures

##### Attention

*Auditory sustained attention test (Murphy et al., 2014)*. This test was developed using E-Prime Professional Software. The duration of the test was approximately 10 min, and the test consisted of 398 trials. In each trial, a digit (from 1 to 7) was presented acoustically and the participants were instructed to press a button as quickly as possible each time they heard either of the digits 1 or 5 through their headphones. In the British version of this test, the digits were presented in English, while in the Brazilian version they were presented in Portuguese. This was the only difference between both English and the Brazilian version of this test. The stimuli were presented diotically at a comfortable listening level that corresponded to a sound pressure level of 70 dB (A). The digit was presented during the first 500 ms of the trial and was followed by an inter-stimulus interval of 1,000 ms. Therefore, digits were presented at a rate of 1 digit/1,500 ms. The target signal probability was 0.28. Two outcome measures were analyzed: correct detection (HIT) and reaction time (RT) for correct detection.

##### Short-term memory

*Visual digit span/forward recall (Murphy et al., 2014)*. This task was developed using E-Prime Professional Software. The digit span task began with a sequence of three digits and allowed 12 attempts per series. The children were instructed to verbally repeat the sequence of numbers after viewing the numbers on a computer screen during each attempt. If performance exceeded 50% (i.e., more than six correct attempts within a series), the number of digits in the sequence was gradually increased. The outcome measure was span performance, i.e., length of the last series completed with greater than 50% accuracy. The exact same version of the test was applied for both groups.

#### Auditory Sensory Measure

##### Auditory processing

*Time compressed speech test*. This test assesses speech intelligibility using compressed stimuli. It has been used to assess temporal acuity in relation to speech intelligibility, specifically the temporal resolution of phonetic information. (Wilson et al., 1994). The performance on this test is correlated to the ability to identify speech spoken at faster rates (Gordon-Salant et al., 2014). Comparing to the other auditory temporal tests, it involves a simpler task in terms of cognitive demand, enabling its use in children under 7 years old. The version applied to the British group was The Tonal and Speech Materials for Auditory Perceptual Assessment, Disc 2.0. Although it is American, it is the most common version used in the United Kingdom. Its Brazilian version, including similar characteristics, was applied to the Brazilian group (Rabelo and Schochat, 2007). In both versions, a list of 50 monosyllabic words was presented; in the American version, the words were 45% compressed, while in the Brazilian version, they were 70% compressed. Both degrees of compressions were clinically recommended by previous studies (Bellis, 2003; Rabelo and Schochat, 2007). The words were presented using a laptop computer (Windows 7 operating system, DirectSound driver) and Trust HS-4100 USB headphones. The stimuli were presented diotically. The outcome measure was the percentage of correctly identified words.

#### Language Measures

##### Phonological processing

*Phonological processing test*. A phonological assessment battery was applied to investigate different levels of phonological awareness in each group. It included simple tasks involving Rhyme and Alliteration detection and also more complex tasks involving phoneme manipulation. For the British group, Rhyme, Alliteration, and Spoonerism subtests of the Phonological Assessment Battery/PhAB (Frederickson et al., 1997) were used. For the Brazilian group, Rhyme, Alliteration and Phonemic manipulation tasks of the Brazilian test PCF (Prova de Consciência Fonológica) were included (Capovilla and Capovilla, 1998). Rhyme and alliteration tasks involve detecting words sharing a common feature or sound, at the end, and at the beginning of the word, respectively. The outcome measure for all subtests was the percentage of correct items.

##### Reading

*Word reading test*. The word reading task involved reading orally and quickly as many words as possible from a word list. This same type of reading task was also used in previous studies addressing orthography’s influence in word reading predictors (Ziegler et al., 2010; Caravolas et al., 2012; Bar-Kochva and Breznitz, 2014). For the British group, the skill was assessed through the subtest *Sight Word Efficiency* of the Test of Word Reading Efficiency – Second Edition (TOWRE – 2, Torgesen et al., 2012). For the Brazilian group, the Single Word Reading Test, developed by Salles, 2001, was applied. Both tests included words varying in terms of regularity (regular and irregular words), length (short and long stimuli), and familiarity (frequent and non-frequent words). The outcome measure was the number of correctly read words. All types of errors, such as omissions, substitutions, inversions, word stress, etc. as well as refusal to read a specific word were considered as a mistake.

For both groups, the tests were carried out in a quiet room, in the school, during the regular school time. The data were collected in one single session of approximately 40 min for each child.

### Statistical Analysis

A-priori sample size analysis indicated that a sample size of around 50 participants would be sufficient to detect a significant interaction effect (large effect size) in the final step of the hierarchical regression with a power of 0.80 and an alpha of 0.05. Intra-group analyses were performed to determine the strength of the association between reading, phonological skills and the other tests in each group. Partial correlation (Pearson) was calculated between all tests in each group, followed by hierarchical regression. Details about these analyses are described in the results section.

The details are described in the next section.

## Results

### Correlation Between Measures in Both Groups

Table 2 demonstrates the performance of each group for all measures. The comparison between groups was not one the purposes of this study; this data was only included to facilitate further discussion regarding intergroup analyses.

**TABLE 2:** Performance of both groups in each of the tests applied.

	British group (*n* = 42)		Brazilian group (*n* = 58)	
	Mean ± *SD*	Median	Mean ± *SD*	Median
**Attention skills**				
HIT (%)	90.7 ± 6	93.6	68.1 ± 15	71.0
Reaction time (ms)	1074 ± 79	1028.5	834 ± 75	832.0
**Short-term memory**			3.95 ± 0.8	
Digit span	4.14 ± 1	5.0	69.33 ± 27	4.0
**Phonological skills**				
Rhyme and alliteration (%)	67.90 ± 28	80.9	69.33 ± 27	75.0
Phoneme task (%)	68.53 ± 30	80.0	24.29 ± 31	6.25
**Reading**				
Single words (%)	53.5 ± 23	64.0	58.5 ± 36	73.0
**Auditory Processing**				
Time compressed speech (%)	83.6 ± 11	88.0	68 ± 6.3	70.0

The correlation between the measures was assessed partialling out the effect of age. The correlation matrix for the British group is demonstrated in Table 3. Reading was significantly correlated with both phonological measures [strong for Rhyme and Alliteration (*r* = 0.687, *p* < 0.001) and moderate for Spoonerism (*r* = 0.489, *p* = 0.015)] and the Digit span task (moderate, *r* = 0.540, *p* = 0.006). The correlation between reading and attention HIT was near significant (*p* = 0.08) and, according to predictions with larger samples sizes, it could be significant; however, the strength of the correlation would still be weak. The phonological skills were correlated not only with each other (moderate, *r* = 0.437, *p* = 0.033), but also with different measures. While Rhyme and Alliteration was correlated with Digit span (moderate, *r* = 0.531, *p* = 0.008) and Time Compressed Speech (moderate, *r* = 0.410, *p* = 0.04), Spoonerism was correlated with Attention HIT (moderate, *r* = 0.426, *p* = 0.038).

**TABLE 3:** Correlations between main measures for the British group.

No.	Measure	1	2	3	4	5	6
**(1)**	Reading	−					
**(2)**	Rhyme and alliteration	0.687**	−				
**(3)**	Spoonerism	0.489*	0.437*	−			
**(4)**	Attention HIT	0.360	0.304	0.426*	−		
**(5)**	Attention reaction time	–0.340	–0.400	–0.152	−0.428*	−	
**(6)**	Digit span	0.540**	0.531**	0.191	0.282	–0.202	−
**(7)**	Time compressed speech	0.241	0.410*	0.121	0.016	–0.285	0.318

*p < 0.05, **p < 0.01.

Table 4 shows the correlation matrix for the Brazilian group. Similarly to the British group, reading was also correlated with both phonological skills [moderate for Rhyme and Alliteration (*r* = 0.455, *p* = 0.001) and moderate for Phoneme manipulation (*r* = 0.433, *p* = 0.002)] and the digit span task (moderate, *r* = 0.435, *p* = 0.002); however, the correlation between Reading and Rhyme and Alliteration was weaker for this group. Phonological skills were strongly correlated with each other (*r* = 0.647, *p* = 0.001). In addition, Rhyme and Alliteration was also correlated to Attention HIT (moderate, *r* = 0.435, *p* = 0.002) and digit span task (weak, *r* = 0.341, *p* = 0.018), while Phoneme manipulation was correlated to digit span only (weak, *r* = 0.398, *p* = 0.005).

**TABLE 4:** Correlations between main measures for the Brazilian group.

No.	Measure	1	2	3	4	5	6
**(1)**	Reading	−					
**(2)**	Rhyme and alliteration	0.455**	−				
**(3)**	Phoneme manipulation	0.433**	0.647**	−			
**(4)**	Attention HIT	0.193	0.435**	0.245	−		
**(5)**	Attention reaction time	0.139	–0.033	0.014	–0.170	−	
**(6)**	Digit span	0.435**	0.341*	0.398**	–0.138	0.119	−
**(7)**	Time compressed speech	0.079	0.164	0.050	0.218	–0.059	0.115

*p < 0.05, **p < 0.01.

### Hierarchical Regression

Hierarchical regression was performed to investigate the relative contribution of each one of those variables to the variance in reading and phonological task performance. For the investigation of reading predictors, the variables were entered, following the specific sequence: the factor age was entered at Step 1, followed by both phonological variables at Step 2, cognitive measures at Step 3 and the auditory sensory measure at Step 4.

Table 5 provides the results of the regression models conducted for the investigation of reading predictors. At Step 1, a significant model was produced for both groups and the contribution to the variance in scores was 43% for the British group and 31% for the Brazilian group. Adding both phonological measures in Step 2, produced a significant *R*^2^ change for both groups, which was explained by Rhyme and Alliteration in both groups [British group (Beta: 0.539); Brazilian group (Beta: 0.231)]. The effect size attributable to phonological measures’ addition to the first model was large to the British group (*f*^2^ = 0.72) and medium to the Brazilian group (*f*^2^ = 0.24). Adding the cognitive measures in Step 3, produced a significant *R*^2^ change for the British group, explained by the Digit span measure (Beta: 0.390), with a small effect size (*f*^2^ = 0.19). For the Brazilian group, considering the minimal effect size found (*f*^2^ = 0.03), a significant change (*p* = 0.05), with a power of 0.80, could be observed only with a sample size of 461 participants in this group. No significant *R*^2^ change was observed with the inclusion of the Time compressed speech measure in Step 4, for either group. In fact, the *R*^2^ change was negative in both groups, which indicates that this variable did not contribute at all to the variance, and this lack of significance wouldn’t change with a more significant number of participants. Thus, the Model 3 was the best one to explain the variance in scores for the British group (72%), with age, Rhyme and Alliteration and digit span as significant contributors. In the Brazilian group, Model 2 was the best one (45%), with age and Rhyme and Alliteration as significant contributors.

**TABLE 5:** Four model for prediction of reading in both groups.

	British group			Brazilian group		
Independent variables	*B*	*SE*	β	*B*	*SE*	β
**Model 1**						
Age	7.98	1.56	0.67**	26.54	5.09	0.57**
*R*^2^	0.43**			0.315**		
**Model 2**						
Age	5.33	1.30	0.45**	19.91	4.92	0.42**
Rhyme and alliteration	0.36	0.07	0.53**	4.04	2.31	0.23*
Phoneme	0.01	0.07	0.01	1.68	1.03	0.22
*R*^2^	0.67**			0.451**		
Δ*R*^2^	0.24**			0.153**		
**Model 3**						
Age	3.57	1.51	0.30*	16.84	5.49	0.36**
Rhyme and alliteration	0.24	0.08	0.36**	3.14	2.45	0.18
Phoneme	0.02	0.07	0.02	1.22	1.05	0.16
Digit span	5.69	2.03	0.39*	9.08	5.22	0.20
Attention HIT	–0.50	0.50	–0.14	0.21	0.29	0.08
Attention RT	–0.02	0.03	–0.10	0.05	0.05	0.11
*R*^2^	0.723**			0.467**		
Δ*R*^2^	0.07*			0.04		
**Model 4**						
Age	3.37	1.54	0.28*	16.49	5.54	0.35**
Rhyme and alliteration	.23	0.08	0.35*	3.25	2.47	0.18*
Phoneme	0.01	0.07	0.02	1.13	1.06	0.15
Digit span	5.18	2.12	0.35*	9.73	5.32	0.22
Attention HIT	–0.41	0.51	–0.11	0.26	0.30	0.11
Attention RT	–0.22	0.03	–0.91	0.26	0.30	0.11
Time compressed speech	0.13	0.15	0.09	–0.42	0.58	–0.74
*R*^2^	0.721**			0.462**		
Δ*R*^2^	–0.007			–0.005		

*p < 0.05, **p < 0.01.

Given the importance of the Rhyme and Alliteration task to reading in both groups, the underlying processes related to this phonological skill were also investigated. The variables were entered following the specific sequence: age at Step 1, followed by cognitive measures in Step 2 and the auditory sensory measure in Step 3. The results are provided in Table 6. At Step 1, a significant model was produced for both groups, that explained 13% of the variance in scores for the British group and 6% in the Brazilian group. Adding the cognitive measures in Step 2, produced a significant *R*^2^ change for both groups, leading to 36% for the British group, with a nearly large effect size (*f*^2^ = 0.36), and 28% for the Brazilian group with a medium effect size (*f*^2^ = 0.30). Digit span was the only significant variable added for the British group (Beta: 0.60), while both Digit span (Beta: 0.39), and Attention HIT (Beta: 0.457) were significant in the Brazilian group. No significant *R*^2^ change was observed with the inclusion of the Time compressed speech measure in Step 3, for either group and, as observed for reading, the raw *R*^2^ change was negative. Thus, the Model 2 was the best model to predict Rhyme and Alliteration skills in both groups, with digit span as the contributor in the British group and both digit span and attention in the Brazilian group.

**TABLE 6:** Three models for prediction of rhyme and alliteration in both groups.

	British group			Brazilian group		
Independent variables	*B*	*SE*	β	*B*	*SE*	β
**Model 1**						
Age	7.08	2.87	0.40*	0.66	0.34	0.25*
*R*^2^	0.13*			0.06*		
**Model 2**						
Age	–0.91	3.42	–0.05	–0.25	0.35	–0.09
Digit span	13.15	3.85	0.60**	0.97	0.30	0.39**
Attention HIT	0.51	1.11	0.098	0.06	0.01	0.45**
Attention RT	–0.03	0.08	–0.097	–0.00	0.00	–0.02
*R*^2^	0.36**			0.28**		
ΔR^2^	0.27**			0.27**		
**Model 3**						
Age	–1.21	3.49	–0.07	–0.25	0.36	–0.09
Digit span	12.21	4.21	0.56**	0.97	0.31	0.38**
Attention HIT	0.62	1.14	0.12	0.06	0.01	0.45**
Attention RT	–0.03	0.08	–0.08	–0.00	0.00	–0.01
Time compressed speech	0.20	0.33	0.10	0.00	0.03	0.00
*R*^2^	0.34**			0.27**		
Δ*R*^2^	0.00			0.00		

*p < 0.05, **p < 0.01.

## Discussion

This research aimed to investigate the word reading predictors in a group of Brazilian and British typically developing children. The overall results demonstrated that Rhyme and Alliteration predicted reading in both groups, with a stronger correlation in the British group. Short-term memory also correlated with reading in both groups; however, it predicted reading only in the British group. Rhyme and Alliteration, on the other hand, correlated with short-term memory in both groups, and also attention in the Brazilian group.

### Reading and Phonological Skills

The correlation between phonological skills and reading, in both groups, was not surprising since there is considerable evidence that phonological processing is universally related to word reading skills (Schneider et al., 1997; Ziegler and Goswami, 2005; Goswami, 2008; Landerl and Wimmer, 2008; Smythe et al., 2008). Both phonological skills involve manipulating phonemes, which is intrinsically related, to some extent, to both orthographies, as they are both alphabetic, i.e., they rely on the principle of basic sounds being represented by letter symbols (Smythe et al., 2008).

Rhyme and Alliteration was the only phonological skill that predicted reading in both groups, indicating that rhyming awareness is an important contributor to successful literacy learning in both languages. However, it was strongly correlated with reading in the British group. This result is in line with the idea that languages lie at different positions along a transparency continuum (Ziegler et al., 2010). Although English has more complex phonological syllable structures and is less transparent than Portuguese, Portuguese is still not completely transparent as Finish and Hungarian, for instance, at the opposite end of the spectrum. Thus, the use of reading strategies, such as the ones involving Rhyme, are important in both languages; however, it is mainly important when learning to read in English, the least transparent orthography. This result corroborates previous studies that demonstrated the importance of this specific phonological skill on the acquisition of the English language, specifically (Bradley and Bryant, 1983; Goswami and Bryant, 1990) as children are encouraged to develop the rhyme analogy strategy involving orthographic chunks, for example when learning to read (Ziegler and Goswami, 2005). Although correlated with reading in both groups, phoneme skills were not as significant as Rhyme and Alliteration, maybe because it assessed the most advanced level of phonological awareness in terms of the level of complexity. Thus, it might not be extensively used as Rhyme and Alliteration when children start learning to read. Also, the lower performance of the Brazilian group compared to the British group might indicate that, although the skill also relates to reading in Portuguese, the task itself was unfamiliar for Brazilian children, maybe because Brazilian teachers don’t use those complex tasks in class as part of the phonic method.

The underlying processes involving Rhyme and Alliteration was also investigated. Short-term memory was a predictor in both groups, as well as attention in the Brazilian group. There was also some degree of correlation with Time Compressed speech in the British group. This last correlation is probably related to the auditory discrimination of those phonological units within each word. The fact that this correlation has been found only in the British group might indicate how significant speech intelligibility is when manipulating Rhyme in English, precisely, probably because of the complexity of the syllables. Also, it suggests an association between this skill and the temporal acuity as the auditory test involved speech compressed in time. Goswami et al. (2002) have reported that children with dyslexia have perceptual deficits involving temporal processing, such as rhythm detection and rapid spectrotemporal integration, as also previously reported by Tallal (1980). Interestingly, she demonstrated that rhythm detection is related to the phonological processing at the syllable level, specifically, acting as a non-speech mechanism used to isolate the suprasegmental attributes of the speech, such as segregating syllable onsets and rhymes. Attention skills was also a predictor, but only in the Brazilian group. According to our initial hypothesis, this result was surprising as we were expecting an even stronger correlation of attention and phonological awareness in the British group. This lack of correlation might be due to a ceiling effect since this group scored more than 90% of correct answers in the attention test, possibly affecting the observation of significant correlations. On the other hand, the correlation found in the Brazilian group corroborated previous studies that have also associated specific attention measures, such as inattentive behavior, with overall phonological awareness tasks (Dally, 2006; De Groot et al., 2017).

### Reading and Cognitive Skills

The results showed that short-term memory and word reading are, to some extent, correlated in both groups, especially in the British group. It is also shown that, when controlling for phonological skills (and its underlying memory demanding), word reading was still predicted by short-term memory in the British group, indicating a direct relationship. This result confirmed that the act of reading single words itself in English also taps on the ability to store information for a short period. This current result not only corroborated previous studies showing the direct contribution of short-term memory to word reading (Mann and Liberman, 1984; Hoien-Tengesdal and Tonnessen, 2011; Kim et al., 2018) but went beyond when it indicated that this relationship probably varies as a function of phonological structure and orthography. As observed by Kim et al. (2018), word reading requires temporary storage to manage phonological, semantic, and orthographic information. This would explain why short-term memory is overall important for word reading in different languages, but it would also explain why it is a direct predictor for languages in which phonological and orthographic information are quite complex, such as in English. Several studies have also associated short-term memory and reading in different languages, such as English, Portuguese, and Chinese (Swanson and Howell, 2001; Engel de Abreu et al., 2014; Kim et al., 2018); however, there was no investigation regarding the direct relationship between word reading and short-term memory. Although the current results have shown promising results regarding this relationship in the British group, further studies should examine this link considering the small effect size observed.

Attention was not a word reading predictor in either group. However, it predicted Rhyme and Alliteration in the Brazilian group, as previously mentioned. Emerging theories associating reading and overall attention skills have been considered (Shaywitz and Shaywitz, 2008), especially through studies addressing the high comorbidity rate of dyslexia and attention-deficit/hyperactivity disorder (Willcutt et al., 2000; Aaron et al., 2002). Sims and Lonigan (2013) investigated the relationship between reading and sustained attention specifically, which was the subcomponent of attention assessed in the current study. They reported that inattentive behavior, when measured by Continuous Performance test, was linked to early reading skills in young children, corroborating the idea that attention plays an important role in the development of early reading skills. In the current study, the results reported for the Brazilian group would also confirm the relevance of attention skills for reading only in terms of phonological awareness. Further studies should investigate the effect of different types of attention skills on word reading.

### Reading and the Auditory Sensory Measure

The auditory sensory measure was not a predictor for reading in either group. In fact, it only correlated with Rhyme and Alliteration tasks in the British group, as already discussed. Conflicting results have been reported regarding the association between reading and auditory sensory processing, not only because of the presence (Tallal, 1980; Habib, 2000; Van Ingelghem et al., 2001) or lack of association between those skills (Nittrouer, 1999; Rosen and Manganari, 2001; Paul et al., 2006) but because of the genuine causal relationship between them (Protopapas, 2013). Protopapas (2013), for instance, raised questions regarding the specific auditory psychoacoustic tasks applied since these are quite complex in terms of cognitive demand, especially when they involve associating the stimuli with a motor or verbal response. To avoid this interference, mainly because we tested young children from 5 years old, we used a quite simple test in terms of cognitive demand, which required repetition of the single words. However, the current test involved temporal degradation of speech, imposing significant linguistic demand, which is an aspect usually controlled in most of the studies through tests, including non-verbal stimuli. Therefore, the current study suggests that the auditory temporal aspect, when assessed with reduced interference of cognitive aspects but high influence of linguistic aspects, has no association with word reading, and this seems to occur regardless of orthography. Despite that, when dealing with phonological awareness, more specifically, Rhyme and Alliteration, the perception and discrimination of individual sound elements in syllables and their segregation, probably demand higher levels or more refined skills involving auditory temporal perception and this demand seems to be higher in English, justifying the correlation found only in the British group.

Overall, the main findings of this study suggest that the relative importance of word reading predictors seems to vary according to language complexity, highlighting the importance of considering particular phonological and orthographic features of each language. The less transparent the orthography, the higher the importance of factors such as phonological awareness, short-term memory, and to some extent, auditory sensory processing skills. This should be taken into consideration when reading assessments and interventions are developed and adapted between countries. This study has some limitations. The sample size was relatively small; thus, the current results should be confirmed with larger sample sizes. In addition, both groups were composed of children from different age ranges, which could have affected the investigation of word reading predictors in different reading stages. There was also one single task for each skill, hence affecting the discussion regarding the influence of task characteristics. Furthermore, as any other cross-linguistic study, variables such as the educational system and culture might have also influenced the results.

## Data Availability Statement

The raw data supporting the conclusions of this article will be made available by the authors, without undue reservation, to any qualified researcher.

## Ethics Statement

The studies involving human participants were reviewed and approved by UCL Research Ethics Committee under protocol number 6688/001. Written informed consent to participate in this study was provided by the participants’ legal guardian/next of kin.

## Author Contributions

CM and D-EB designed the experiments. CM performed the experiments and analyzed the data. CM, ES, and D-EB wrote the manuscript. All authors contributed to the article and approved the submitted version.

## Conflict of Interest

The authors declare that the research was conducted in the absence of any commercial or financial relationships that could be construed as a potential conflict of interest.
